# Comparative Analysis of Sugar Metabolites and Their Transporters in Sugarcane Following *Sugarcane mosaic virus* (SCMV) Infection

**DOI:** 10.3390/ijms222413574

**Published:** 2021-12-17

**Authors:** Sehrish Akbar, Wei Yao, Lifang Qin, Yuan Yuan, Charles A. Powell, Baoshan Chen, Muqing Zhang

**Affiliations:** 1 Guangxi Key Laboratory for Sugarcane Biology & State Key Laboratory for Conservation and Utilization of Agro Bioresources, Guangxi University, Nanning 530005, China; sehrishakbar2050@yahoo.com (S.A.); yaowei@gxu.edu.cn (W.Y.); lifangqin@st.gxu.edu.cn (L.Q.); yuany@st.gxu.edu.cn (Y.Y.); chenyaoj@gxu.edu.cn (B.C.); 2 IRREC-IFAS, University of Florida, Fort Pierce, FL 34945, USA; capowell@ufl.edu

**Keywords:** sugar metabolites, sugar transporter, transcriptomics, proteomics, *Sugarcane mosaic virus* (SCMV), sugarcane

## Abstract

*Sugarcane mosaic virus* (SCMV) is one of the major pathogens of sugarcane. SCMV infection causes dynamic changes in plant cells, including decreased photosynthetic rate, respiration, and sugar metabolism. To understand the basics of pathogenicity mechanism, we performed transcriptome and proteomics analysis in two sugarcane genotypes (Badila: susceptible to SCMV and B-48: SCMV resistant). Using *Saccharum spontaneum* L. genome as a reference, we identified the differentially expressed genes (DEGs) and differentially expressed proteins (DEPs) that participate in sugar metabolism, transport of their metabolites, and Carbohydrate Activating enZYmes (CAZymes). Sequencing data revealed 287 DEGs directly or indirectly involved in sugar metabolism, transport, and storage, while 323 DEGs are associated with CAZymes. Significant upregulation of glucose, sucrose, fructose, starch, and SWEET-related transcripts was observed in the Badila after infection of SCMV. B-48 showed resistance against SCMV with a limited number of sugar transcripts up-regulation at the post-infection stage. For CAZymes, only glycosyltransferase (GT)1 and glycosyl hydrolase (GH)17 were upregulated in B-48. Regulation of DEGs was analyzed at the proteomics level as well. Starch, fructose, glucose, GT1, and GH17 transcripts were expressed at the post-translational level. We verified our transcriptomic results with proteomics and qPCR data. Comprehensively, this study proved that Badila upregulated sugar metabolizing and transporting transcripts and proteins, which enhance virus multiplication and infectionl.

## 1. Introduction

Sugarcane (*Saccharum officinarium* L.), belonging to the genus *Saccharum*, is a semi-perennial grass grown in warm temperate and tropical climates [1,2]. Sugarcane is a commercially important crop because of its ability to produce high sugar yield and other biomolecules. Sugarcane produces approximately 85% of the total sugar [3,4]. According to an estimate, Brazil is the biggest producer of sugarcane, producing approximately 37% of the total world production in 2019, followed by India, China, Thailand, and Pakistan, accounting for 18.7%, 10.8%, 5.2%, and 3.3% production, respectively [5].

Increased sugar production is due to its capacity to store sucrose molecules in stem parenchyma and culm internodes [6]. Sucrose is the most common form of sugar in plants, and its metabolism is divided into three basic steps: biosynthesis, transportation, and storage [7]. Sucrose is synthesized in the chloroplast of leaf cells during the Calvin cycle of photosynthesis.

Sucrose is released into the cytosol by sucrose transporting enzymes termed sucrose-6-phosphate phosphohydrolase (S6PP), also known as sucrose phosphate phosphatase (EC 3.1.3.24). S6PP dephosphorylates sucrose-6-phospahte (Suc6P) and releases free sucrose molecules [7,8]. Free sucrose molecules then turn towards phloem vessels to translocate themselves from source to sink and other organs of plants. After transportation from photosynthetic tissues, the fate of sucrose molecules is to store themselves into the vacuoles of parenchymatous cells of non-photosynthetic tissues [9,10]. Sucrose molecules adopted both symplastic and apoplastic routes for their entry into vacuoles. Sucrose synthase enzyme (SuSy) (EC 2.4.1.13) catalyzes the reversible conversion reaction from sucrose into UDP-glucose, depending upon plant requirement. Additionally, the sucrose phosphate synthase enzyme (SPS) (EC 2.4.1.14) activates the sucrose molecules to generate Suc6P, releasing energy by dephosphorylation through the S6PP enzyme. Sucrose molecules utilize this energy to enter the vacuole, which is finally stored as glucose and fructose [11].

Sucrose transporter facilitates the sucrose transport from one cell to another cell over the apoplast. Lignified fiber cells surrounding the vascular bundles play an essential role in sucrose transport [12]. These layers block the solutes for apoplastic movement when sucrose accumulation starts [13]. Sucrose transporter gene families in sugarcane are not entirely known due to the highly complex, polyploid, and heterozygous genome. However, the sucrose transporter 1 (SUT1) group has been isolated from bundle sheath cells of mature internodes and mature leaves of sugarcane [14,15,16]. At the same time, the SUT4 group has been characterized by internodes [17]. Another critical enzyme involved in the sucrose synthesis cycle is sucrose phosphate synthase II (SPSII), essential for sugar metabolism, sucrose synthesis, and sucrose storage [18,19,20].

Sugar production and availability are the major determining factors of plant growth and are affected by various biotic and abiotic factors. Extensive research has been performed to understand biotic factors such as fungi and bacteria on sugar availability. However, very little is known about the effect of virus infection on sugar production and its availability [21,22,23]. Until now, virus infection regulates cellular machinery to utilize the plant cellular functions and resources for survival and multiplication. Infected plant cells are turned into the metabolic sink. Carbohydrate allocation and signaling are altered [21,24]. This shifting reprogrammed primary metabolisms such as changes in carbohydrates, amino acids, and lipids levels that result in accumulation of sugars and starch, increase in respiration rate and decline in photosynthetic activity [21,24].

Plant pathogens produce a variety of Carbohydrate-Active enZYmes (CAZymes), which are responsible for biosynthesis and breakdown of the cell wall and are involved in host-pathogen interaction. CAZymes are classified into four functional groups: glycosyltransferases (GTs) (EC 2.4), glycoside hydrolases (GHs) (EC 3.2), carbohydrate esterases (CEs) (EC 3.1), and polysaccharide lyases (PLs) (EC 4.2) [25,26]. The GTs formed glycosyl bonds between sugar molecules, responsible for cellulose and hemicellulose production, signaling, and defense mechanism [27,28,29,30]. GHs break the glycosyl bonds between the sugar molecules, responsible for modification of cell wall and abscission [25,31]. PLs are involved in the cleavage of glycosidic bonds for modification and breakdown of pectin [32,33]. The CEs performed the de-acetylation of sidechains of polysaccharides and crossed linked lignin with hemicellulose [34,35]. CBM is another non-enzymatic class that allows modification and specific binding with carbohydrate biopolymers [36,37].

Previous studies on understanding the molecular mechanism of plant-virus interactions were primarily performed in dicotyledonous model plants such as *Nicotiana* and *Arabidopsis*. However, the physiological response of monocots under viral infection is not clearly understood [38]. Therefore, the interaction analysis of *Sugarcane mosaic virus* (SCMV) (family: Potyviridae) was carried out on sugar metabolites, transporters, and CAZymes in sugarcane. Previously, we generated resistant genotype (B-48) using coat protein (CP) of SCMV [39]. B-48 transgenic line showed maximum yield, high photosynthetic efficiency, and defense response against SCMV [39,40,41]. Therefore, this study aimed to perform the extensive transcriptomics and proteomics analysis of SCMV infected and non-infected sugarcane leaves of two contrasting genotypes (B-48: resistant and Badila: wild type). We explored that SCMV infection altered sugar metabolites, resulting in high sucrose content, glucose level, and starch molecules in the Badila genotype. B-48 showed reduced sugar metabolites and their transporters accumulation at transcriptome and proteome levels.

## 2. Results

### 2.1. Analysis of Sugarcane Transcriptome following SCMV Infection

Sugarcane transcriptome was compared after the infection treatment between SCMV and mock treatment plants on wild type (Badila) and resistant genotype (B-48). A fully expanded leaf was collected from each genotype and subjected to microscopic observation. We characterized the morphological changes post-SCMV infection, which depicted that B-48 showed vigorous growth and healthy leaves compared to the confinement of virus particles in Badila [40].

From each infected and non-infected leaf sample, RNA-seq libraries were constructed and sequenced through Illumina sequencing. On average, 30 million reads per library were obtained, which were mapped to the *Saccharum spontaneum* L. genome due to the unavailability of the *Saccharum officinarum* genome [41]. The *S. spontaneum* genome comprises eight chromosomes with eight alleles (A, B, C, D, E, F, G, and H) [42]. We estimated approximately 69–74% sequence alignment with the reference genome (Supplementary Table S1). Through the eggNOG (e-mapper) online tool, we annotated 1.1 × 10^4^ differentially expressed genes (DEGs). The number of DEGs associated with sugar metabolism were identified and involved in the transport of sugar metabolites from total DEGs. A total of 287 DEGs were directly or indirectly involved in sugar synthesis, breakdown, and transport of its metabolites (Supplementary Table S2).

### 2.2. Effect of SCMV Infection on Sugar Metabolism

**Figure 1:**
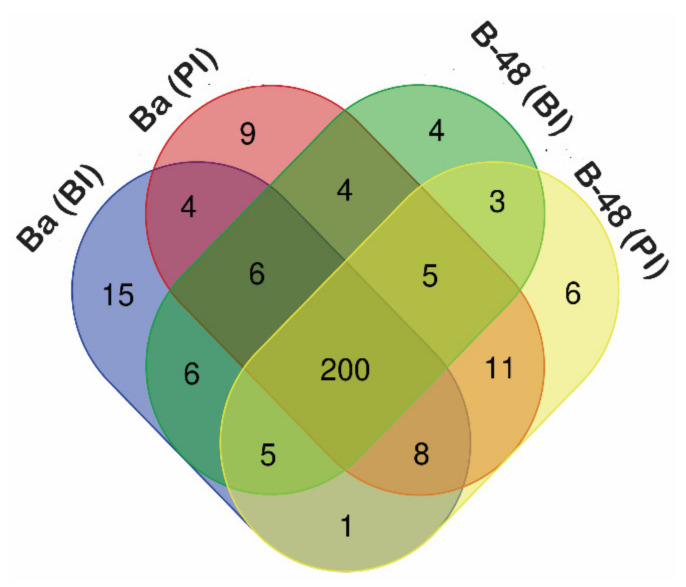
Number of common and unique transcripts associated with sugar metabolites and their transporters in Badila and B-48 genotypes, before and post SCMV infection.

During viral infections, infected plant cells are utilized as nitrogen and carbon sources to synthesize new molecules. Infection reduces photosynthetic activity, increases respiration rate, and increases the demand for photosynthetic assimilates [24]. Previously, some viral infections such as *Tomato mosaic virus* (ToMV) and *Cauliflower mosaic virus* (CaMV) caused the abundant accumulation of sucrose and sugars in the plant cells [43,44,45]. Consistent with previous findings, 287 DEGs were identified to be involved in sugar metabolism following the SCMV infection in B-48 and Badila (Figure 1).

About 200 (70%) DEGs are shared in both genotypes before and after virus infection. However, 16 (5.5%) unique DEGs showed increased accumulation in Badila before infection (BI), while 20 (0.069%) unique DEGs were enhanced in Badila post-infection (PI). Moreover, some of the DEGs were upregulated in the B-48 genotype before the infection of the virus. We identified 20 (0.069%) unique DEGs in B-48 (BI), while 26 (0.09%) DEGs were highly expressed only in B-48 (PI).

### 2.3. Differential Gene Expression Profiling of Sugar Metabolites Transcripts following SCMV Infection

A comparative gene expression analysis was performed to decipher the effect of SCMV infection on the changes in sugar level between Badila and B-48 and to highlight the sugar metabolizing DEGs. The changes in the expression level of transcripts were identified to be involved in primary metabolism to produce sucrose and starch. It has been postulated that virus infection causes an increase in sucrose, glucose, starch, and other energy-producing molecules [46]. Inside the infected cell, sugar level profoundly affected the synthesis of storage molecules, development of symptoms, and defense response [46]. It eventually resulted in acclimation of metabolic rate to overcome the increased demand of energy for virus multiplication and defense response to combat virus replication [24].

#### 2.3.1. Fructose

**Figure 2:**
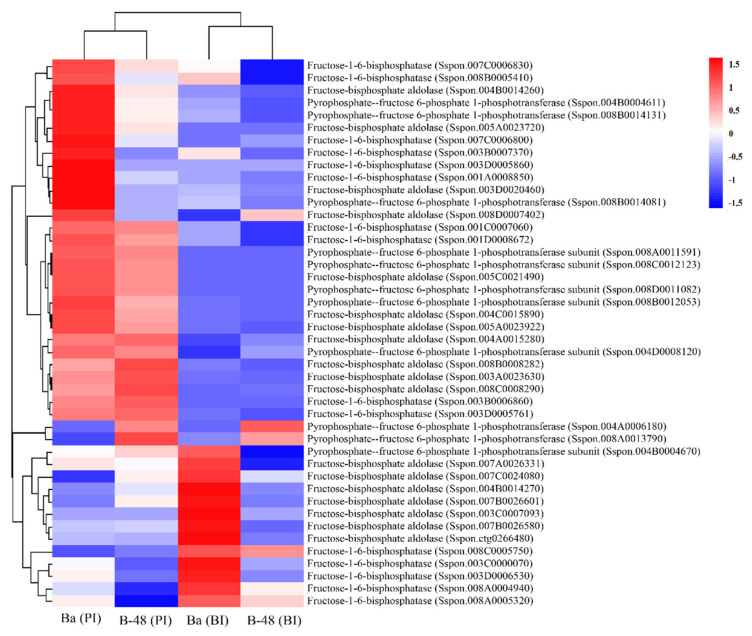
Differential gene expression involved in sugar metabolism in Ba (BI), Ba (PI), B-48 (BI), and B-48 (PI). The number of transcripts involved in fructose synthesis. Upregulated DEGs are shown in red color while down-regulated DEGs are in blue. Ba (BI): Badila (Before Infection); Ba (PI): Badila (Post Infection); B-48 (BI): B-48 (Before Infection); B-48 (PI): B-48 (Post Infection).

Virus replication demands an enormous amount of energy and synthesis of new biomolecules and thus strongly relies on the plant metabolic machinery. Overexpression of sugar metabolizing transcripts generates enhanced energy. About 44 DEGs related to fructose production were systematically analyzed for gene expression (Figure 2). Interestingly, about 200 (70%) DEGs are common in both genotypes, before and after virus infection. However, 16 (5.5%) unique DEGs showed increased accumulation in Badila before infection (BI), while 20 (0.069%) unique DEGs were enhanced in Badila post-infection (PI). Moreover, some of the DEGs were upregulated in the B-48 genotype before the infection of the virus. We identified 20 (0.069%) unique DEGs in B-48 (BI), while 26 (0.09%) DEGs were highly expressed only in B-48 (PI).

Badila maximized the expression of 13 DEGs before infection of the virus, while 29 DEGs were upregulated under SCMV infection. Among these upregulated DEGs, ten fructose 1,6 biphosphate (FBP) and three pyrophosphate fructose 6-phosphate-1 phosphotransferase (PFP) (EC: 2.7.1.90) subunit transcripts were enhanced before infection. However, post-infection enhanced ten transcripts of both FBP and fructose biphosphate aldolase (aldolase) (EC 4.1.2.13) and nine transcripts of the PFP subunit. Four transcripts were upregulated in B-48 before infection of SCMV, including two FBP and two PFP subunits. However, four transcripts of FBP, eight transcripts of PFP subunit, and six of aldolase were directly increased in the B-48 genotype post-SCMV infection.

#### 2.3.2. Starch

**Figure 3:**
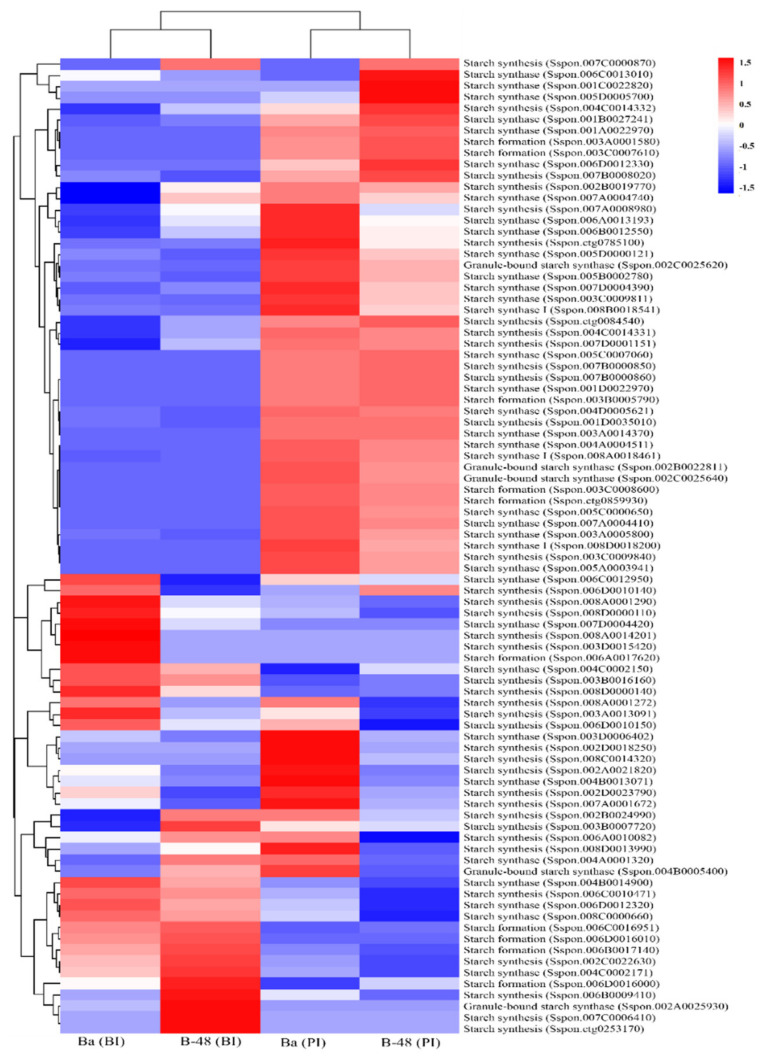
Differential gene expression involved in sugar metabolism in Ba (BI), Ba (PI), B-48 (BI), and B-48 (PI). The number of transcripts associated with starch synthesis. Upregulated DEGs are shown in red color while down-regulated DEGs are in blue. Ba (BI): Badila (Before Infection); Ba (PI): Badila (Post Infection); B-48 (BI): B-48 (Before Infection); B-48 (PI): B-48 (Post Infection).

Virus infection leads to the deposition of starch molecules in the chloroplast of infected cells [47,48,49,50]. We have investigated the expression pattern of transcripts related to starch synthesis under SCMV infection (Figure 3). Eighty-seven transcripts related to starch synthetase (EC 2.4.1.21) were highlighted in agreement with this role. Twenty transcripts were upregulated before virus infection in Badila, while after infection, fifty-nine transcripts were enhanced in Badila. The expression pattern of Badila depicted that SCMV infection increased the metabolic rate and starch accumulation. However, 20 transcripts showed maximum expression before infection in B-48, while 43 transcripts were observed increased expression in B-48 post virus infection.

#### 2.3.3. Sucrose

**Figure 4:**
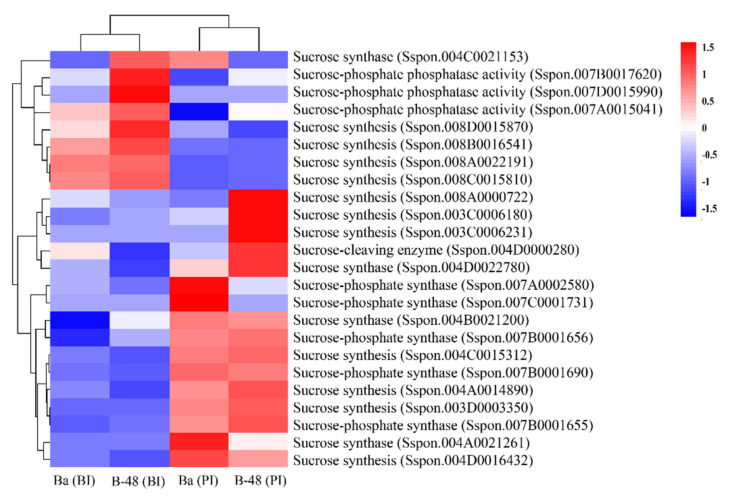
Differential gene expression involved in sugar metabolism in Ba (BI), Ba (PI), B-48 (BI), and B-48 (PI). Number of DEGs related to sucrose metabolites. Upregulated DEGs are shown in red color while down-regulated DEGs are in blue. Ba (BI): Badila (Before Infection); Ba (PI): Badila (Post Infection); B-48 (BI): B-48 (Before Infection); B-48 (PI): B-48 (Post Infection).

Plant viral infections accumulate sucrose in the leaves of infected cells [21]. Twenty-four DEGs were identified related to sucrose production at the transcript level (Figure 4). Five sucrose related DEGs were upregulated in Ba (BI), of which four are sucrose cleaving enzymes, while one is involved in sucrose phosphate phosphatase (SPP) (EC. 3.1.3.24) activity. Noticeably, SCMV infection enhanced 13 DEGs in Badila, four of which were related to sucrose synthase (SuSy) (EC 2.4.1.13), five were sucrose phosphate synthase (SPS) (EC 2.4.1.14), and four were involved in sucrose production. However, B-48 resistant genotype over-expressed eight DEGs before SCMV infection, of which three were related to SPP activity, the other three were sucrose cleaving enzymes, and one was SuSy. B-48 upregulated 13 DEGs post-SCMV infection, of which three were SPS, while other ten ones were related to SuSy.

#### 2.3.4. Glucose

**Figure 5:**
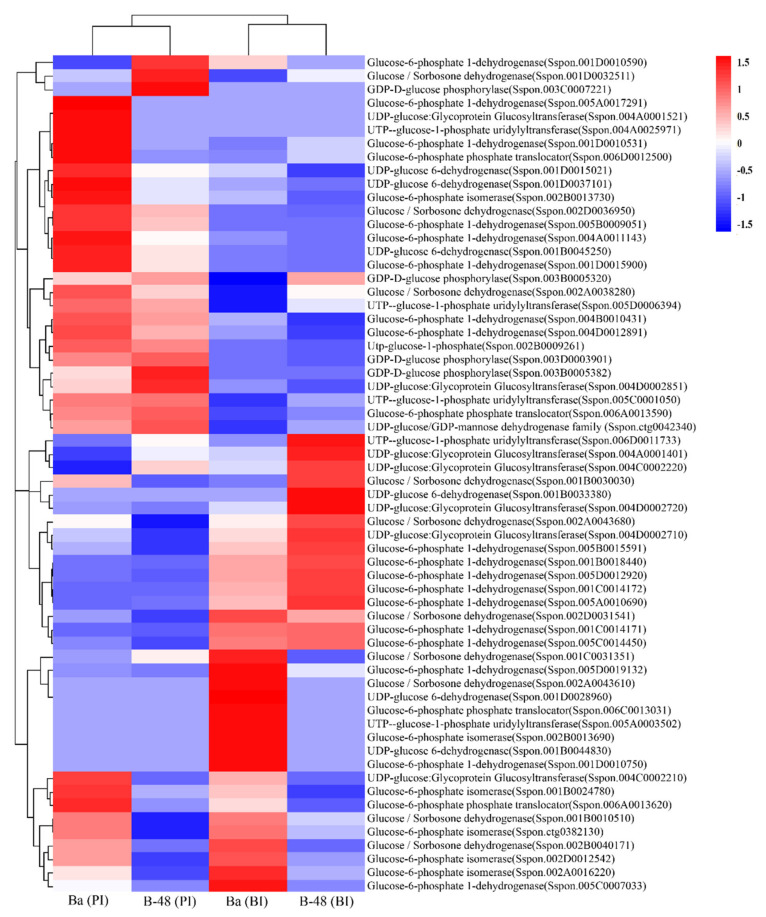
Differential gene expression involved in sugar metabolism in Ba (BI), Ba (PI), B-48 (BI), and B-48 (PI). Number of DEGs linked with glucose production. Upregulated DEGs are shown in red color while down-regulated DEGs are in blue. Ba (BI): Badila (Before Infection); Ba (PI): Badila (Post Infection); B-48 (BI): B-48 (Before Infection); B-48 (PI): B-48 (Post Infection).

Plant virus interaction causes profound perturbations in the glucose level [24]. Intriguingly, 24 transcripts directly or indirectly involved in glucose production showed increase expression in Badila (BI) (Figure 5). These transcripts were glucose-6-phosphate-1-dehydrogenase (G6PD) (EC 1.1.1.49) (8 DEGs), glucose sorbosone dehydrogenase (GSDH) (EC 1.1.99.32) (5 DEGs), UDP-glucose-6-dehydrogenase (UGDH) (EC 1.1.1.22) (2 DEGs), UTP glucose-1-phosphate uridylyltransferase (UGPUT) (EC 2.7.7.9) (1 DEG), UDP glucose glycoprotein glycosyltransferase (UGGT) (EC 2.4.1) (1 DEG), glucose-6-phosphate isomerase (GPI) (EC 5.3.1.9) (5 DEGs) and glucose-6-phosphate phosphate translocator (GPT2) (2 DEGs). SCMV infection modulated 32 transcripts in Badila, including ten G6PD, two UGGT, five UGPUT, one GPT2, eight GPI, three UGDH, and three GDP-D-glucose phosphorylases (GDPGP1) (EC 2.7.7.78). SCMV resistant B-48 expressed 16 DEGs before inoculation of the virus, including GSDH (4 DEGs), UGDH (1 DEG), UGPUT (1DEG), UGGT (3 DEGs) and GPI (7 DEGs). Post-infection upregulated 20 transcripts in B-48, including G6PD (6 DEGs), GSDH (3 DEGs), GDPGP1 (4 DEGs), GPI (1 DEG), UGDH (1 DEG), UGPUT (3 DEGs), UGGT (1 DEG) and GPT2 (1 DEG).

### 2.4. Differential Gene Expression Profiling of Sugar Transporter Transcripts following SCMV Infection

**Figure 6:**
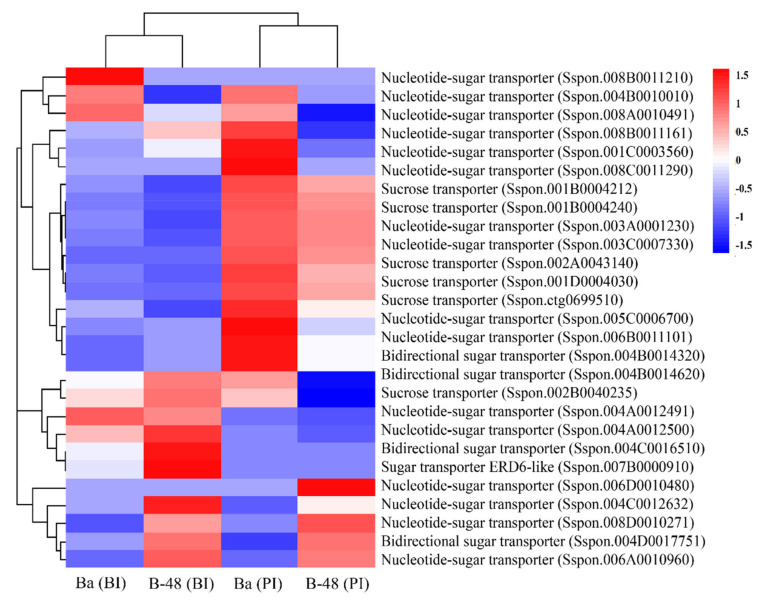
Differential expression analysis of sugar transporter DEGs in Ba (BI), Ba (PI), B-48 (BI), and B-48 (PI). Upregulated DEGs are shown in red color while down-regulated DEGs are in blue. Ba (BI): Badila (Before Infection); Ba (PI): Badila (Post Infection); B-48 (BI): B-48 (Before Infection); B-48 (PI): B-48 (Post Infection).

Pathogen attack hijacks the plant sucrose transporters, increasing sugar efflux at the infection site, ultimately resulting in pathogen growth and plant defense [23,51]. The number of transcripts were determined to be involved in the sugars-will-eventually-be-exported-transporters (SWEET) and sugar transport (Figure 6).

In total, 45 DEGs were identified involved in the transport of sugar and its metabolites. A high level of transcripts was expressed in Badila (PI). Differential gene expression analysis depicted that six DEGs involved in sucrose transport, while 22 ones were associated with nucleotide-sugar transport. Six DEGs involved in sucrose and nucleotide sugar transport were upregulated in before-inoculation of Badila (BI). Interestingly, eighteen DEGs were highly expressed in post-inoculation of Badila (PI), of which four were involved in sucrose transport while fourteen were associated with sugar-nucleotide transport. However, the B-48 resistant genotype elevated a constant level of expression before and post-inoculation. B-48 (BI) enhanced eleven transcripts, including two involved in sucrose transport, nine associated with sugar-nucleotide transport. Similarly, 11 DEGs were upregulated in B-48 (PI), including five related to sucrose transport, six involved in nucleotide sugar transport.

### 2.5. Identification of Carbohydrate Active enZYmes (CAZy) in Badila and B-48

Different families of CAZymes are identified in the genus *Saccharum* which contains 20 GHs, 14 GTs, 1 PL), 1 AA, 4 CBM, and 1 CE (www.cazy.org.com, accessed on 1 November 2021). The highest number of transcripts were identified from the GH28 family, which comprises polygalacturonase. GH17 was the second most abundant hydrolase family found in the RNA seq data of both genotypes. Consistent with previous findings, GH28 was enriched in young leaves while GH17 was most frequent in the cell wall of sugarcane [52]. Glycosyltransferase (GT) 1 was the only GT found in our study which participated in polysaccharide synthesis [53].

**Figure 7:**
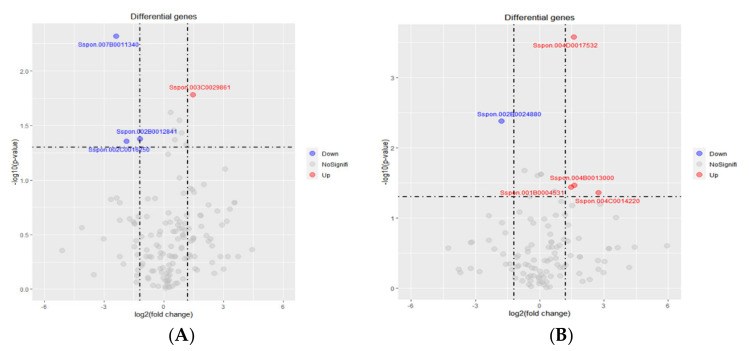
Number of transcripts of transcripts of CAZymes genes in Badila (**A**) and B-48 (**B**), before and post SCMV infection. Differential gene expression was considered as significant at FC > 2 and *p*-value < 0.05.

DEGs analysis showed that 145 genes associated with CAZymes were expressed in Badila under SCMV infection. Among them, only one DEG (Sspon.003C0029861) was up-regulated, while three DEGs (Sspon.007B0011340, Sspon.002B0012841, Sspon.002C0016250) were down-regulated. However, the majority of them showed no significant expression (Figure 7A). GT1 was up-regulated DEG in Badila, while one DEG of GH17 and two of GH28 were down-regulated.

In B-48, differential gene expression analysis depicted that SCMV infection perturbed 110 DEGs associated with CAZymes. Among these DEGs, four were up-regulated (Sspon.004D0017532, Sspon. 004B0013000, Sspon. 001B0004531, Sspon.004C0014220) and one (Sspon.002B0024880) was down-regulated, while rest of 105 DEGs showed no significant differential response (Figure 7B). Up-regulated DEGs in B-48 were belonged to families GT1 and GH17, while down-regulated DEG was from the GH28 family.

### 2.6. Validation of Transcriptome Profiling through Quantitative Real-Time PCR

**Figure 8:**
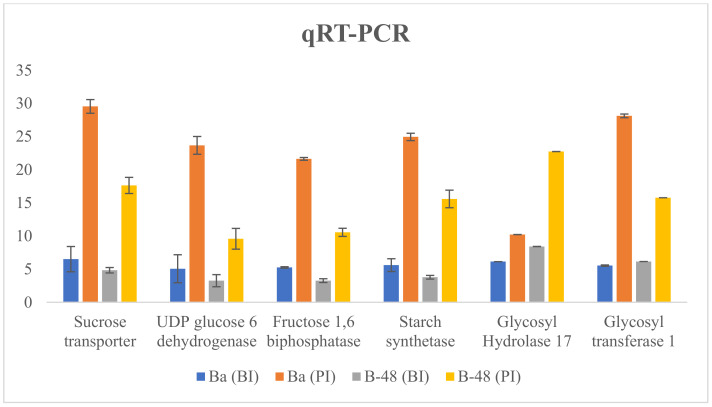
Confirmation of expression analysis through qRT-PCR. *Y*-axis determined expression value while *X*-axis represents the selected transcripts. Standard deviation is estimated through error bars. For each sample, triplicates were used for analysis (*p* < 0.05). Ba (BI): Badila (Before Infection); Ba (PI): Badila (Post Infection); B-48 (BI): B-48 (Before Infection); B-48 (PI): B-48 (Post Infection).

Upregulated expression pattern of selected DEGs was confirmed using qRT-PCR (Figure 8). qPCR data depicted that SCMV infection regulated genes related to sugar metabolism, its metabolites transporters and CAZymes. Interestingly, Badila had a much higher expression pattern of sugar metabolite genes and its transporter than B-48. It is noticeable that B-48 (PI) also enhanced gene expression levels related to sugar metabolites to fulfill the increased demand of energy to combat virus infection. Expression level of CAZymes (GH17 and GT1) were monitored in Badila and B-48 genotypes, which highlighted significant expression level. GH17 expression level was increased in B-48 after SCMV infection. However, GT1 showed higher expression in Badila genotype post SCMV infection. Our qPCR data of few selected genes such as sucrose transport, UDP-glucose 6 dehydrogenase, starch synthesis, fructose 1,6 biphosphatase, glycosyl hydrolase 17 and glycosyl transferase 1 showed consistency with RNA-seq analysis data.

### 2.7. Protein Expression Profiling

**Figure 9:**
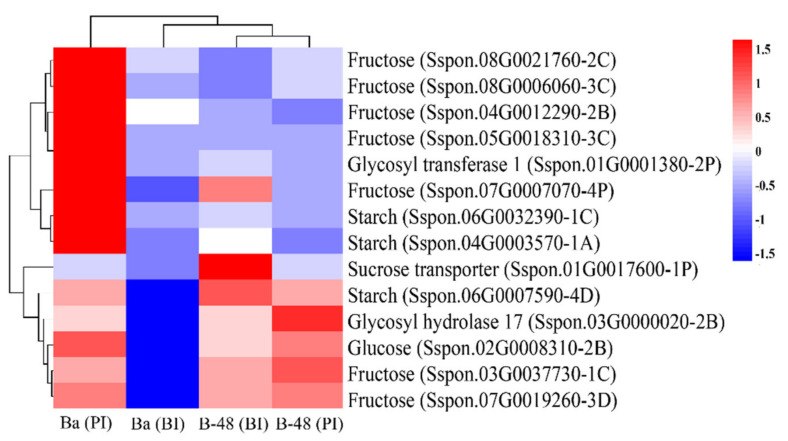
Differential abundance analysis of proteins involved in sugar metabolites and their transporters. Upregulated DEPs are shown in red color while down-regulated DEPs in blue color. Ba (BI): Badila (Before Infection); Ba (PI): Badila (Post Infection); B-48 (BI): B-48 (Before Infection); B-48 (PI): B-48 (Post Infection).

Protein functional data were analyzed to identify the abundance of protein and their consistency of abundance at the translational level. Overall, thirty-seven proteins were related to sugar metabolites, transporters and CAZymes with differential abundance patterns. Here, we highlighted the expression pattern of highly abundant proteins (Figure 9).

Interestingly, our transcriptomics and proteomics data showed consistency at the transcriptional and post-transcriptional levels. Transcripts related to glucose, starch, fructose, GH17, and GT1 were also highly abundant at the protein level. We have noticed that many transcripts were expressed at the RNA level, but only a few are regulated at the protein level. It could be because only a few intense transcriptional changes could express at the protein level [54]. Among the up-regulated DEPs, one protein was involved in sucrose transport; sixteen were associated with fructose, four were related to starch synthesis, six corresponded to glucose, and ten were related to different CAZymes. We highlighted only fourteen highly differentiating proteins. Badila enhanced protein abundance level only after SCMV infection. Highly abundant DEPs are related to fructose, starch, glucose, fructose, and GT1. In contrast, two fructose, one starch, one glucose, and one GH17 was highly abundant in B-48, post-SCMV infection. The abundance of proteins was found to be consistent with RNA seq data.

**Figure 10:**
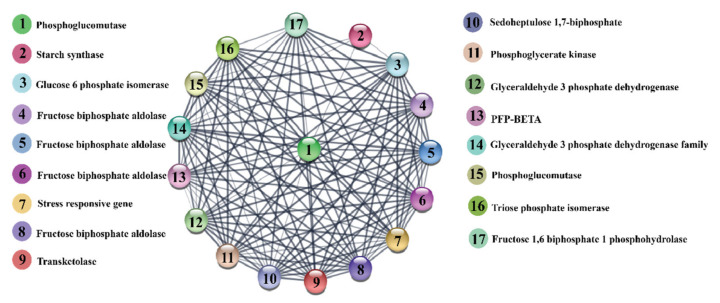
Protein–protein interaction network of differentially expressed proteins (DEPs) of sugar metabolites and their associated enzymes in Badila and B-48 genotypes. Proteins are represented through nodes and links between proteins are indicated by lines.

Next, we mapped the protein–protein interaction of differentially abundant proteins related to sugar metabolites, transporter proteins, and CAZymes. Total 17 proteins related to 225 edges were bound together with a confidence level of 0.40. Selected DEPs were shown to be strongly connected. As it has been postulated that proteins mainly operate in complexes [55]. All these proteins are directly or indirectly linked, showing the dependent relationship with each other (Figure 10).

## 3. Discussion

Sugar production is essential in plants during photosynthesis, and the sugar level modulates internal mechanisms and environmental responses that regulate growth and development [56,57,58]. The previous investigation discovers the molecular mechanism of sugar sensing and plant signaling [58]. Stress stimuli such as bacterial, fungal, and viral infections can regulate the source-sink relationship. This stimulus also modifies the sugar level in the infected cells, leading to the activation of various independent signaling pathways [59,60]. It is noticeable that sugars enhance the expression of pathogenesis-related (PR) genes [61,62], lipoxygenase genes, and wound inducible proteinase inhibitor II [63,64]. The underlying mechanism in sugar signaling pathways in plants has been investigated, revealing various sugar-related genes regulated at the transcriptional level. However, little information is available regarding the transcriptional and post-transcriptional mechanisms underlying these processes. Here, we explored the sugar metabolites, their transporter genes, and Carbohydrate Activating enZYmes (CAZymes) that trigger the diverse signaling pathway. Intriguingly, signaling pathways stimulate different reactions (i) initiation of plant defense response in resistant cultivar; (ii) utilization of energy resources for virus multiplication in the susceptible cultivar.

Sucrose is the major photo-assimilate produced during photosynthesis and is one of the leading signaling molecules in plant cells [65,66]. Sucrose synthetase (SuSy) is a crucial glycosyltransferase enzyme involved in sugar metabolism [67]. This enzyme is responsible for sucrose synthesis from fructose and UDP-glucose and sucrose cleavage into fructose and UDP-glucose, utilizing UDP [67]. Sucrose phosphate phosphatase (SPP) is another significant enzyme responsible for sucrose synthesis by dephosphorylating sucrose phosphate [68,69,70]. Interestingly, we have observed that Badila and B-48 expressed a high number of SuSy and SPS transcripts. Post-SCMV infection data showed that both genotypes enhanced the expression of SuSy and SPS DEGs. However, comparative enhancement of expression was higher in Badila as compared to B-48. Our result was consistent with previous findings, in which SCMV infection decreased SPS levels in infected sugarcane leaves [71]. Additionally, Shalitin and Wolf reported that the *Cucumber mosaic virus* (CMV) infection on melon caused downregulation of SPS and sucrose accumulation in leaves [72].

SuSy has been reported to be involved in the conversion of sucrose to starch molecules. SuSy catalyzes the cleavage of sucrose molecules to produce ADP-G, which is translocated to the chloroplast from cytosol for starch synthesis [73]. Moreover, low SuSy activity reduced starch accumulation in potato tubers, maize endosperm, and carrot roots [74,75,76]. SuSy genes were linked with starch accumulation and storage pathways. Interestingly, during viral multiplication, SuSy, soluble sugars, and starch levels were elevated in the leaves [21,46,77]. Our expression pattern data showed high starch synthesis-related transcript in Badila after SCMV inoculation.

Upon infection, viral RNA synthesis occurred from intermediates of photosynthesis, oxidative pentose phosphate pathway (PPP), and the precursor generated from degraded nucleotides of host RNA molecules [78,79,80]. Glucose-6-phosphate dehydrogenase (G6PD) is the crucial enzyme in PPP, which generates purine and pyrimidine nucleotides [81]. Earlier studies highlighted the role of G6PD in local and systemic virus multiplication. The current study determined the upregulation of G6PD expression in Badila (BI) leaves. It is consistent with previous studies, in which an increase in G6PD was noticed in the mesophyll protoplasts of tobacco leaves after the infection with various viruses, including *Potato virus* Y (PVY), *Potato virus* X (PVX), *Cucumber mosaic virus* (CMV), *Turnip mosaic virus* (TuMV) and *Tobacco rattle virus* (TRV) [82]. Another significant enzyme is glucose-6-phosphate isomerase (GPI) that generates energy from glycolysis and gluconeogenesis pathways [83]. It is also involved in PPP [84] and starch synthesis [85]. Significant upregulation of GPI transcripts was noticed in Badila (PI), suggesting a possible involvement in starch synthesis. Another signaling molecule, fructose, depicted a differential expression response in the infected sugarcane leaves. Fructose 1,6-biphosphate (FBP), pyrophosphate fructose 6-phosphate-1-phosphotransferase (PFP), and aldolase expression levels were higher post-SCMV infection in both genotypes. Same as the previous, *Mal de Río Cuarto virus* (MRCV) infection in wheat leaves causes an imbalance in carbohydrates metabolites level. Infected leaves showed a notable increase in glucose, fructose, starch, and sucrose level at 21 days post-inoculation (dpi) [46].

Sugar transporters believe in playing a significant role in pathogen infection [86]. Up till now, multiple sugar transporters have been identified. Sucrose transporters (SUTs) are first reported transporters, then SWEET and Sugar Transport Proteins (STPs) were identified [87,88,89,90]. It has been postulated that SWEET transporters are upregulated during pathogen infection [91]. These transporters expel sugars into extracellular spaces outside cells. Pathogens feed on this exported sugar and replicate [92]. Similarly, these transporters also translocate sugars to the infected cells to initiate host defense mechanisms [93]. Intriguingly, susceptible Badila cultivar in our study enhanced multiple transporter transcripts supporting the hypothesis that sugars were fuel for virus replication.

Carbohydrate-active enzymes (CAZymes) were reported to be involved in the metabolism of oligosaccharides, polysaccharides, and glycoconjugates inside plant cells [94]. While for plant pathogens, CAZymes take part in the degradation of the host’s cell walls and storage compounds [94]. Here, we performed the in silico investigation of CAZymes from the genomes of Badila and B-48 genotypes. CAZymes are classified as glycoside hydrolases, glycoside transferases, polysaccharide lyases, and carbohydrate esterases [95]. Glycosyltransferase (GT) family 1 is the most prominent in plants involved in the glycosylation step of the triterpenoid biosynthesis pathway and synthesis of plant defense molecules such as anthocyanins phenolics, salicylates, and glucosinolates [96,97,98]. We have identified the up-regulation of GT1 DEGs in both Badila and B-48 genotypes. Badila genotype enhanced the GT1 expression even at the protein level. Previously, it has been investigated that phenylpropanoids glycosylation in tobacco plants plays a significant role in *Tobacco mosaic virus* infection [99]. Consistently, Badila (PI) showed high expression of GT1 at transcriptome as well as proteome level. Another family of CAZymes is GH, which is responsible for the hydrolysis of bonds between two carbohydrates, between carbohydrate and protein, or between carbohydrate and lipids [100]. They have multiple functions, one of which is the defense response against pathogen attack [31]. Among the total 386 identified differentially expressed genes of CAZymes, GH17 is highly expressed in B-48 (PI) while it is down-regulated in the Badila genotype. GH17 gene expression is very intense, which expresses itself at the protein level. Previous studies supported the constitutive expression of GH17 in tomato decreased virulence against *Potato virus* X by releasing damage-associated molecular patterns (DAMPs), which in turn activates plant immune response [100].

This study provides deep insight into the underlying mechanism of *Sugarcane mosaic virus* (SCMV) symptoms in sugarcane, emphasizing the regulation of sugar metabolites. Comparative transcriptomics and proteomics analysis highlighted the differences between B-48 and Badila. Badila genotype enhanced a higher number of sugar metabolites following viral infection. High sugar metabolites levels and the expression of CAZymes genes in infected tissues increased carbon availability for viral multiplication. B-48 showed a defense response against SCMV through a profound balance between photosynthetic assimilates and respiration rate.

## 4. Materials and Methods

### 4.1. Plant Samples and SCMV Infection

**Figure 11:**
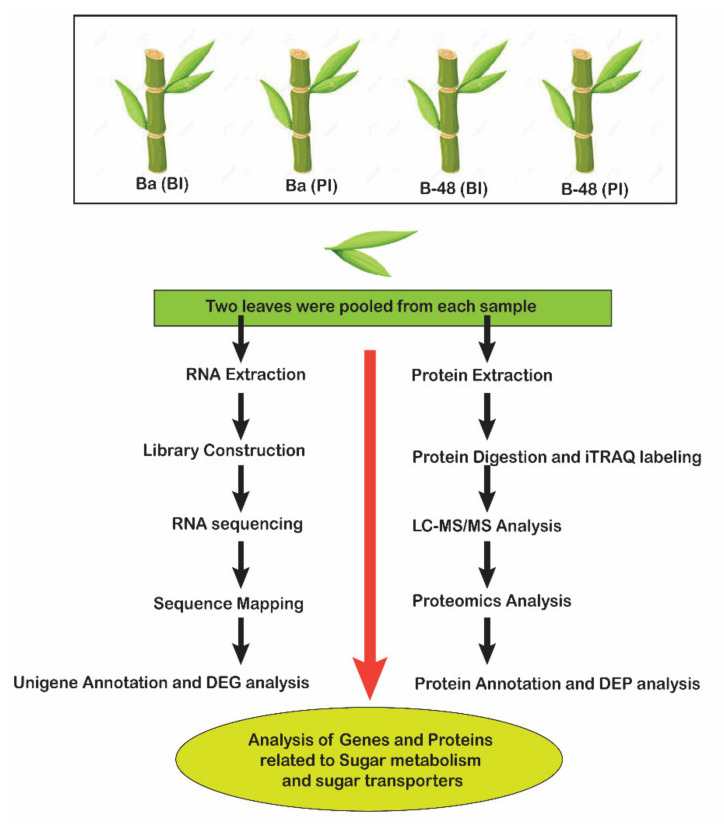
Diagrammatic representation of transcriptomic and proteomics work scheme. Ba (BI): Badila (Before Infection), Ba (PI): Badila Post Infection, B-48 (BI): B-48 (Before Infection), B-48 (PI): B-48 (Post Infection). Two leaf samples were collected from Badila and B-48 before infection of SCMV. Similarly, two leaf samples from Badila and B-48 genotype were collected at 14 days after inoculation (DAI).

Two different genotypes of sugarcane (*Saccharum officinarium* L.) were selected in this study (1) B-48 (SCMV resistant genotype generated by Guangxi University) (2) Badila (SCMV susceptible cultivar). Before mechanical inoculation, two leaf samples were randomly harvested from each genotype. Non-inoculated plants of each genotype were selected as a control plants. Then, mechanical inoculation with sap was performed using the extract of SCMV diseased leaves, as mentioned previously [40]. Inoculation was performed in triplicates. Inoculated plants were kept at 24 °C in the greenhouse with 16 h of light and 8 h of dark for 14 days to develop a systemic infection. Again, two leaves were collected from each genotype from infected plants for further analysis (Figure 11).

### 4.2. Transcriptome Analysis

#### 4.2.1. RNA Extraction

According to the manufacturer’s instructions, RNA extraction from collected leaf samples was performed using TRIzol^®^ Reagent (Thermo Fisher Scientific, Waltham, MA, USA). RNA quantity and quality were measured by NanoDrop 2000 (ThermoScientific, Waltham, MA, USA) and RNA Nano 6000 Assay Kit of the Agilent Bioanalyzer 2100 system (Agilent Technologies, Santa Clara, CA, USA), respectively.

#### 4.2.2. RNA Sequence Library Preparation

The sequencing library was prepared using NEB Next^®^ Ultra^TM^ RNA Library Prep Kit for Illumina and visualized on Agilent 2100 Bioanalyzer (Agilent Technologies, Santa Clara, CA, USA). Each library was sequenced using Illumina HiSeq 2500 platform (Biomarker Technologies, Rohnert Park, CA, USA) to generate paired end reads.

#### 4.2.3. Genome Assembly and Mapping

Sequenced libraries were assembled into a complete genome by mapping with the reference genome of the publicly available sequence of *S. spontaneum* L. [30]. Analysis of raw reads was performed through FastQC (http://www.bioinformatics.babraham.ac.uk/projects/fastqc/, accessed on 8 November 2021). Reads quality was further improved through adapter removal, deletion of low-quality reads, and poly-N sequences.

#### 4.2.4. Functional Annotation of Genes

Functional annotation of genes was searched through KEGG, GO, and eggNOG e-mapper online tool. KEGG functional analysis was performed on KOBAS 2.0 software (http://kobas.cbi.pku.edu.cn/, accessed on 19 October 2021), while for GO enrichment analysis, the R-based program Wallenius non-central hypergeometric distribution program was used. DESeq R package (1.10.1) was used for differential gene expression analysis. Genes with a False Discovery rate (FDR) of ≤0.05 and an absolute value of log2 Ratio ≥ 1 were considered DEGs.

#### 4.2.5. Real-Time Quantitative PCR Analysis

RNA extraction from leaf samples was performed using TRIzol^®^ Reagent (Thermo Fisher Scientific, Waltham, MA, USA). RNA quality and concentration was calculated by NanoDrop^TM^ 2000/2000c (ThermoScientific, Waltham, MA, USA) spectrophotometer and Agilent RNA 6000 Nano Kit (Agilent Technologies, Santa Clara, CA, USA), respectively. The cDNA synthesis was performed using the 5× Primescript RT master mix (Takara Bio, Shiga, Japan). qPCR reaction mixture comprised of 25 µL master mix containing 12.5 µL TB Green Premix Ex Taq II (Tli RNase H Plus), 1 µL of each primer (10 µM), cDNA (2 µL), and water up to 25 µL. Actin was selected as a reference gene. A real-time PCR system (Light Cycler 96 Roche, Shangai, China) was used with the following thermal profile: initial denaturation 95 °C for 30 s, denaturation at 95 °C for 5 s, then annealing and extension at 60 °C for 30 s. The expression level was statistically analyzed using the 2^−ΔΔC^_q_ formula as described previously in [101], List of primers mentioned in Supplementary Table S4.

### 4.3. Proteomics Analysis

#### 4.3.1. Protein Extraction and Digestion

Protein was extracted from powdered leaf samples using 10% TCA/acetone solution, as described in [40]. Protein concentration was measured using Bradford Protein Assay Kit (SangonBiotec, Shangai, China, C503041) following the manufacturer’s protocol.

For protein digestion, 10 mM DTT was used to reduce 100 µg of extracted protein and then alkylated with 50 mM ammonium bicarbonate buffer. Alkylated protein was digested with 1 mg/mL of trypsin (1:50 *w*/*w*) by overnight incubation at 37 °C. After protein digestion, an equal amount of formic acid (0.1%) was supplemented. Then, peptides were purified on Strata-XC 18 columns after washing with 5% acetonitrile (ACN) solution and eluted further with ACN + 0.1% FA solution. Eluted peptides were dried through Speed Vac and suspended in a 0.5 M TEAB (Tetraethylammonium bromide) solution. The suspension then proceeded for iTRAQ labeling.

#### 4.3.2. Peptide Labeling and Fractionation

Peptide samples were labeled and fractionated using iTRAQ Reagent 6 plex Multiplex Kit (Proteomics Grade AB Sciex, San Francisco, CA, USA) and high pH reverse-phase HPLC (WatersBridge Peptide BEH C18; 130 Å, 3.5 μm, 4.6 × 250 mm), respectively, according to the procedure mentioned in [41]. Samples were vacuum dried and proceeded for MS analysis.

#### 4.3.3. LC-MS/MS Assessment and Data Processing

Labeled samples were assessed using NanoLC 1000 LC-MS/MS, with the same procedure as explained in [41]. MS/MS raw generated data was analyzed with the “Transcriptome translated peptide” database. In addition, Sequest software (Washington, DC, USA) integrated with Proteome discoverer (version 1.3) was used for analysis. Proteins with false discovery rate (FDR) value ≤ 0.01% and *p*-value < 0.05 were considered as significant.

#### 4.3.4. Protein Functional Annotation and Enrichment Analysis

Proteins were functionally annotated by the Kyoto Encyclopedia of Genes and Genomes (KEGG) [102] database, and hierarchical clustering was performed through the “pheatmap” function using R-package. Protein–protein interaction was analyzed by STRING (version 11.5) online database (http://www.string-db.org/, accessed on 4 November 2021). Selected proteins were mapped through STRING database and visualized through Cytoscape (v2.8; http://www.cytoscape.org/, accessed on 4 November 2021).

### 4.4. Carbohydrate Active enZYmes (CAZymes) Detection

CAZymes encoding genes of genus *Saccharum* were predicted using CAZy database classification [99] (www.cazy.org, accessed on 1 November 2021).

## Data Availability

RNA sequencing data can be found in the Short Read Archive at NCBI (https://www.ncbi.nlm.nih.gov/sra, accessed on 11 October 2021) with following accession numbers: B-48 (BI) Replicate 1: SRR10058141, B-48 (BI) Replicate 2: SRR10058140, B-48 (PI) Replicate 1: SRR10058139, B-48 (PI) Replicate 2: SRR10058138, Badila (BI) Replicate 1: SRR10058145, Badila (BI) Replicate 2: SRR10058144, Badila (PI) Replicate 1: SRR10058143, Badila (PI) Replicate 2: SRR10058142.

## Supplementary Materials

The following are available online at https://www.mdpi.com/article/10.3390/ijms222413574/s1.
